# Total Antioxidant Status (TAS), Superoxide Dismutase (SOD), and Glutathione Peroxidase (GPx) in Oropharyngeal Cancer Associated with EBV Infection

**DOI:** 10.1155/2019/5832410

**Published:** 2019-07-08

**Authors:** Małgorzata Strycharz-Dudziak, Małgorzata Kiełczykowska, Bartłomiej Drop, Łukasz Świątek, Ewa Kliszczewska, Irena Musik, Małgorzata Polz-Dacewicz

**Affiliations:** ^1^Chair and Department of Conservative Dentistry with Endodontics, Medical University of Lublin, 20-059 Lublin, Poland; ^2^Department of Medical Chemistry, Medical University of Lublin, 20-059 Lublin, Poland; ^3^Department of Information Technology and Medical Statistics, Medical University of Lublin, 20-059 Lublin, Poland; ^4^Department of Virology, Medical University of Lublin, 20-059 Lublin, Poland

## Abstract

A growing number of studies reveal that oxidative stress is associated with viral infections or cancer development. However, there are few reports assessing the relationships between oxidative stress, viral infection, and carcinogenesis. The present study analyzed the level of total antioxidant status (TAS) as well as the activities of glutathione peroxidase (GPx) and superoxide dismutase (SOD) in patients with oropharyngeal cancer both Epstein-Barr virus (EBV)-positive and EBV-negative in comparison with the control group. The correlations between these parameters and EBV type (wild-type LMP1 (wt-LMP1) or LMP1 with deletion (del-LMP1)), level of antibodies against EBV, the degree of tumor differentiation, and TNM classification were also investigated. Fresh frozen tumor tissue samples collected from 66 patients with oropharyngeal squamous cell carcinoma were tested using nested PCR assay for EBV DNA detection. Spectrophotometric methods were used to measure TAS values as well as SOD and GPx activities in homogenates of tissue, using diagnostic kits produced by Randox Laboratories. Sera from all individuals were investigated using ELISA method to detect the presence of Epstein-Barr virus capsid antigen (EBVCA) IgM and IgG, Epstein-Barr virus nuclear antigen (EBNA) IgG, and early antigen (EA) IgG antibodies. The level of TAS and activities of antioxidant enzymes (GPx and SOD) were significantly decreased in tissues with oropharyngeal cancer, particularly in EBV-positive cases. In 82.3% of patients, wt-LMP1 was detected. Significantly lower TAS, GPx, and SOD values were stated in patients infected with wild-type EBV. The presence of antibodies against early antigen (anti-EA) was detected in over 80% of patients, which suggests reactivation of EBV infection. The correlation between the degree of tumor differentiation and TN classification, especially in EBV-positive patients, was also observed. Determination of these parameters may be useful in evaluating tumor burden in patients with various stages of oropharyngeal cancer and could be an important prognostic factor. Future studies are needed to understand the role of EBV lytic reactivation induced by oxidative stress.

## 1. Introduction

Head and neck cancer (HNC) is a very important global problem. In 2020, HNC is expected to affect approximately 833,000 new patients worldwide and 151,000 in Europe [1]. HNC is a frequent malignancy that mainly develops in the epithelial linings of the oral cavity, oropharynx, hypopharynx, and larynx. Most of the lesions are squamous cell carcinomas (SCCs) traditionally considered as associated with tobacco and alcohol exposure [2]. However, various viruses were also demonstrated to play an important role in the etiology of head and neck SCC.

Epstein-Barr virus (EBV), a member of the *Herpesviridae* family, *Lymphocryptovirus* genus that infects about 95% of adult population all over the world, is the first known human oncogenic virus. EBV is a dsDNA gammaherpesvirus and is associated with Burkitt's lymphomas (BL), Hodgkin's lymphomas (HL), nasopharyngeal cancer (NPC), and gastric carcinomas (GC) [3]. Similar to other herpesviruses, EBV establishes a latent infection periodically reactivated into the lytic cycle which plays an important role in the pathogenesis of EBV-related tumors [4–6]. During latent infection, several specific viral proteins such as EBNA1, EBER1 and 2, and BamHI-A rightward transcripts (BART) as well as latent membrane protein 1 and 2 (LMP1, LMP2) are expressed [7, 8]. The oncogenic role of LMP1 is well established. It was demonstrated that EBV variant with a 30 bp deletion (amino acids 346–355) including part of C terminal activating region 2 isolated from nasopharyngeal tumor had a greater transforming activity than the reference LMP1 [9].

Patients with NPC exhibit an elevated level of antibodies to several EBV antigens, including the viral capsid antigen (VCA), early antigen (EA), and EB nuclear antigen (EBNA) which are very useful in clinical diagnosis [10–14].

Numerous number of studies have shown that EBV infection is associated with the production of ROS and/or activation of ROS-associated signalling pathways [15, 16]. According to some researchers, ROS formation may be induced by LMP1 [15].

Superoxide dismutase (SOD), glutathione peroxidase (GPx), and catalase (CAT) are the three major enzymatic antioxidant defense systems responsible for scavenging free radicals and nascent oxygen [17]. Patel et al. [18] demonstrated the risk of oral cancer development in individuals with lowered activity of antioxidant enzymes.

Superoxide dismutase is a decisive antioxidant enzyme in aerobic cells, which is responsible for the elimination of superoxide radicals. SOD catalyzes the dismutation of two molecules: hydrogen peroxide (H_2_O_2_) and molecular oxygen (O_2_). Glutathione peroxidase (GPx) is a selenocysteine-dependent enzyme. GPx in cells is the most important hydrogen peroxide- (H_2_O_2_-) scavenging enzyme which converts hydrogen peroxide to water [19]. SOD and GPx can directly counterbalance the oxidant attack and protect the cells against DNA damage.

The present study analyzed the level of total antioxidant status (TAS) as well as the activities of GPx and SOD in homogenates of tissue collected from the patients with oropharyngeal cancer both EBV-positive and EBV-negative compared to the control group. The relationship between these parameters and the type of EBV (wild-type LMP1 (wt-LMP1) or deletion 30 bp (del-LMP1)), the level of antibodies against EBV, the degree of tumor differentiation (grading (G)), and the TN classification were also investigated.

## 2. Materials and Methods

### 2.1. Patients

The present study involved 66 patients with a diagnosed and histopathologically confirmed oropharyngeal SCC. The patients were hospitalized at the Otolaryngology Division of the Masovian Specialist Hospital in Radom, Poland. The patients had not received radiotherapy or chemotherapy before. Clinical and epidemiological characteristics of the patients are presented in Table 1.

The tissue samples were collected from all patients during surgery and frozen at −80°C until analysis. TNM classification was determined during primary diagnosis according to the criteria of the Union Against Cancer (UICC) [20]. Histological grading was performed according to the World Health Organization criteria, which divide tumors into three types: well differentiated (G1), moderately differentiated (G2), and poorly differentiated (G3) [21].

#### 2.1.1. Control Group

The control group consisted of 30 patients suitably selected in terms of sex, age, place of residence, smoking, and alcohol consumption, who were hospitalized due to nonneoplastic diseases of the throat and larynx (chronic inflammation of palatine tonsils). Tissue material was collected from these people during surgery and frozen at −80°C until analysis. All persons from the control group were EBV negative. In terms of sociodemographic features, smoking, and alcohol consumption, these groups did not differ, and therefore, the features did not affect the values of examined parameters.

#### 2.1.2. Serum Collection

Venous blood samples collected from the patients and control group were centrifuged at 1500 rpm for 15 min at room temperature, and the serum was frozen at −80°C until analysis.

The research was approved by the Medical University of Lublin Ethics Committee and is in accordance with the GCP regulations (no. KE-0254/133/2013-23.05.2013).

### 2.2. DNA Extraction from Fresh Frozen Tumor Tissue

Fragments of the fresh frozen tumor tissue (20 mg), both from the patients with OSCC and from the control subjects, were cut and homogenized in a manual homogenizer Omni TH (Omni International, Kennesaw, Georgia, USA). DNA was extracted using a protocol as described in the DNeasy Tissue Kit Handbook (Qiagen GmbH, Hilden, Germany). Purified DNA was quantified by spectrophotometry (Epoch Microplate Spectrophotometer, BioTek Instruments Inc., Winooski, Vermont, USA). The isolates were kept at −20°C until the test was conducted. To verify the quality of the obtained DNA (presence of inhibitors of Polymerase Chain Reaction), a *β*-globin assay was performed [22].

#### 2.2.1. Detection of EBV DNA

For EBV DNA detection, all PCR reactions were carried out in the final volume of 25 *μ*l using HotStartTaq DNA Polymerase (Qiagen, Germany). Concentrations of PCR components were prepared as follows: 2.0 mM MgCl_2_, 0.2 mM dNTPs, 0.5 *μ*M of each forward and reverse primers, and 0.5 U of HotStartTaq polymerase. During each run, the samples were tested together with one negative (nuclease-free water) and positive control (EBV-positive cell line, Namalwa, ATCC-CRL-1432) [22].

#### 2.2.2. Genotyping of LMP1

PCR screening for the EBV LMP1 subtype based on exon 3, defined as wild-type (wt-LMP1) or del-LMP1, was done using specific primers: forward 5′-AGC GAC TCT GCT GGA AAT GAT-3′; revers 5′-TGA TTA GCT AAG GCA TTC CCA-3′. Concentrations of PCR components were prepared as follows: 2.0 mM MgCl_2_, 0.2 mM dNTPs, 0.5 *μ*M of each forward and revers primers, and 0.5 U Hot Start DNA polymerase and 5 *μ*l of extracted DNA. The reaction mixture (25 *μ*l) was incubated at 95°C for 15 min, followed by 45 cycles at 94°C for 1 min, 57°C for 1 min, 72°C for 1 min, and a final extension at 72°C for 10 min. PCR products were analyzed by gel electrophoresis in a 3% agarose gel and visualized under UV light [22].

### 2.3. Oxidant Parameters

The tissue samples were rinsed with 0.9% NaCl and stored at –80°C until the analysis. Tissue homogenates (10% *w*/*v*) were prepared in 0.1 mol l–1Tris-HCl buffer, pH = 7.4 using a laboratory MPW-120 homogenizer, and supernatants were obtained by centrifugation at 5000 × g for 30 min.

The following oxidant parameters were determined in homogenates of cancer tissue: total antioxidant status (TAS) values as well as activities of superoxide dismutase (SOD) and glutathione peroxidase (GPx).

TAS values were assayed using diagnostic kit produced by RANDOX (Randox Laboratories Ltd., Crumlin, County Antrim, UK) according to Miller et al. [23] and expressed in mmol of TAS/10 mg of protein.

SOD activity was determined using diagnostic kit RANSOD produced by RANDOX (Randox Laboratories Ltd., Crumlin, County Antrim, UK) according to Arthur and Boyne [24] and expressed in U of SOD/10 mg of protein.

GPx activity was determined using diagnostic kit RANSEL produced by RANDOX (Randox Laboratories Ltd., Crumlin, County Antrim, UK) according to Paglia and Valentine [25] and expressed in U of GPx/mg of protein. Protein was measured using the method of Bradford [26]. The assays were performed with the use of spectrophotometer SPECORD M40 (Carl Zeiss, Jena, Germany).

### 2.4. Serological Tests

To detect antibody levels, serological tests were used with ELISA method. Designed antibodies were as follows: anti-VCA IgM (NovaLisa Epstein-Barr Virus VCA IgM; NovaTec Immundiagnostica GmbH, Germany; catalog number: EBVM0150), anti-VCA IgG (NovaLisa Epstein-Barr Virus VCA IgG; NovaTec Immundiagnostica GmbH, Germany; catalog number: EBVG0150), and anti-EBNA IgG (NovaLisa Epstein-Barr Virus EBNA IgG; NovaTec Immundiagnostica GmbH, Germany; catalog number: EBVG0580), antibodies anti-EA IgG (ELISA-VIDITEST anti-EA (D) EBV IgG; Vidia, Czech Republic; catalog number: ODZ-006). All tests were performed according to the manufacturer's instructions.

The NovaTec Epstein-Barr Virus (EBV) IgG-ELISA is intended for the qualitative determination of IgG class antibodies against Epstein-Barr virus. Samples are considered positive if the absorbance value is higher than 10% over the cut-off. The level of antibodies is expressed as NovaTec-Units = NTU.

ELISA-VIDITEST anti-EA is a semiquantitative test. Samples with absorbances higher than 110% of the cut-off value are considered positive.

### 2.5. Statistical Analysis

Descriptive statistics were used to present patient baseline characteristics. Means and standard deviations (SD) were calculated. For variables with nonnormal distribution, the Mann-Whitney *U* test and Kruskal-Wallis test were used. Pearson's chi-square test was used to investigate the relationship between LMP1 subtype and clinical and demographical parameters. Statistical significance was defined as *p* < 0.05.

## 3. Results

**Figure 1:**
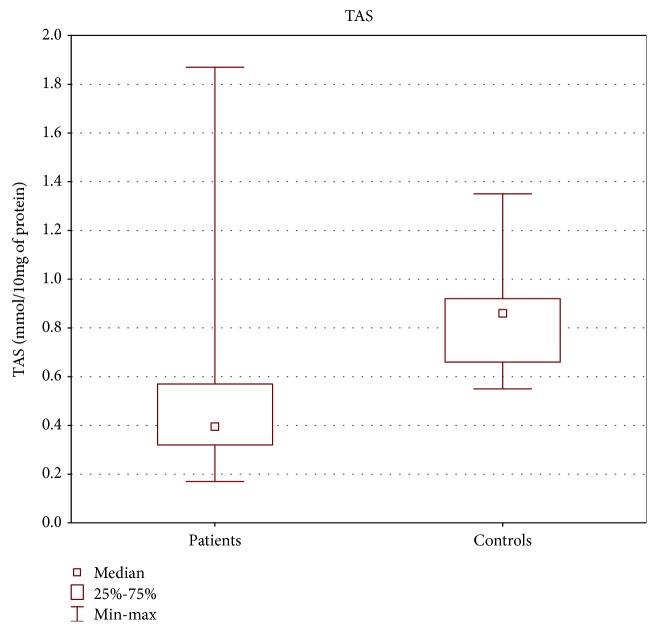
TAS level in tissue of patients with oropharyngeal cancer compared with the control group (mmol/10 mg of protein). Mann-Whitney *U* test; *p* = 10^−6^.

**Figure 2:**
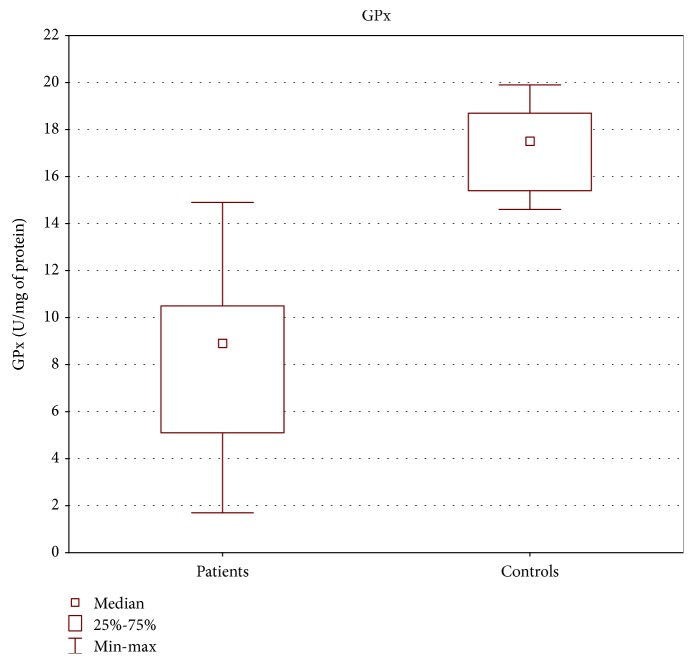
GPx activity in tissue of patients with oropharyngeal cancer compared with the control group (U/mg of protein). Mann-Whitney *U* test; *p* = 10^−6^.

**Figure 3:**
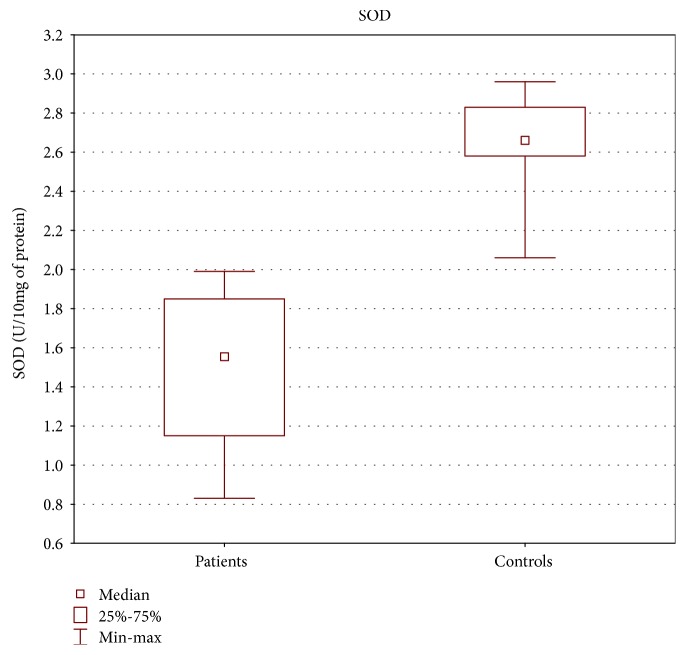
SOD activity in tissue of patients with oropharyngeal cancer compared with the control group (U/10 mg of protein). Mann-Whitney *U* test; *p* = 10^−6^.

TAS level as well as GPx and SOD activities in the tissue of patients with oropharyngeal cancer were statistically lower than those in the control group (Figures 1–3).

Statistically significant differences were stated in TAS, GPx, and SOD values in EBV-positive and EBV-negative patients (Table 2). All analyzed parameters had the lowest values in EBV-positive patients (TAS = 0.38 ± 0.13, GPx = 5.36 ± 2.7, and SOD = 1.12 ± 0.16) and the highest in the control group (TAS = 0.81 ± 0.19, GPx = 17.36 ± 1.7, and SOD = 2.63 ± 0.22).

Moreover, examined parameters of oxidative stress had significantly different values in patients infected with different types of EBV. Significantly lower TAS, GPx, and SOD values were stated in patients infected with wild-type LMP-1 (Table 3).

The analysis of the level of antibodies against EBV revealed that EBVCA and EBNA level was the highest in EBV-positive patients (EBVCA: 72.9 ± 17.7 in EBV-positive vs. 63.4 ± 9.6 in EBV-negative; EBNA: 69.1 ± 15.8 in EBV-positive vs. 60.8 ± 9.4 in EBV-negative patients). Similarly, the highest level of EA was detected only in EBV-positive patients. All differences were statistically significant (Table 4).

**Figure 4:**
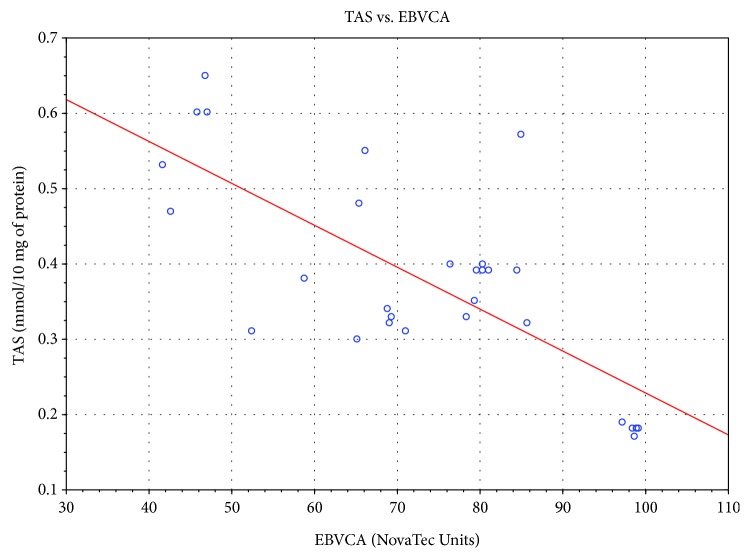
Correlation between tissue level of TAS and serum level of anti-EBVCA. Spearman's rank correlation test; *r* = −0.586596; *p* = 0.0008.

**Figure 5:**
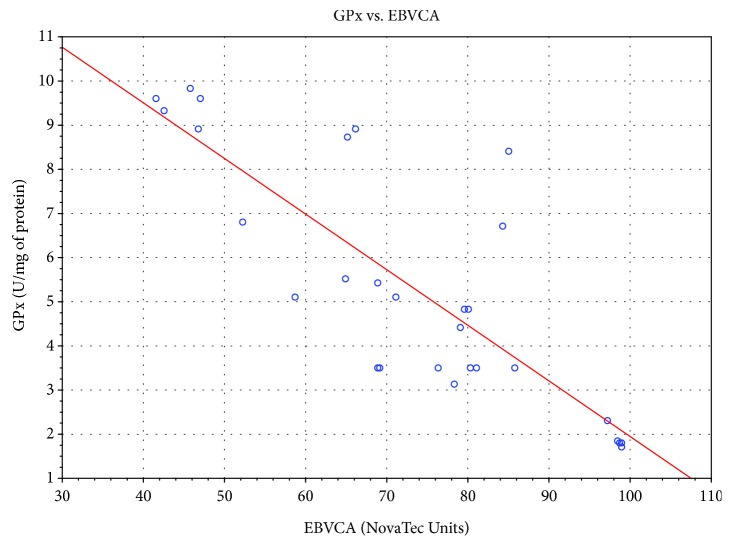
Correlation between tissue level of GPx and serum level of anti-EBVCA. Spearman's rank correlation test; *r* = −0.814861; *p* = 10^−6^.

**Figure 6:**
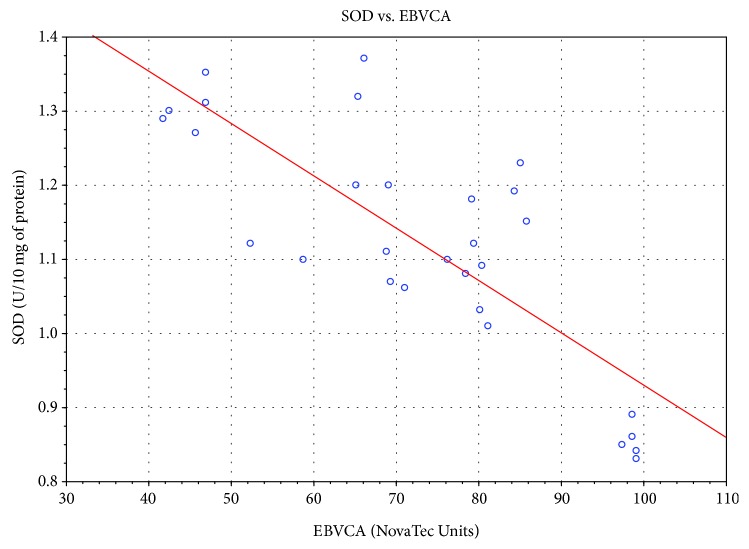
Correlation between tissue level of SOD and serum level of anti-EBVCA. Spearman's rank correlation test; *r* = −0.733276; *p* = 6 × 10^−6^.

**Figure 7:**
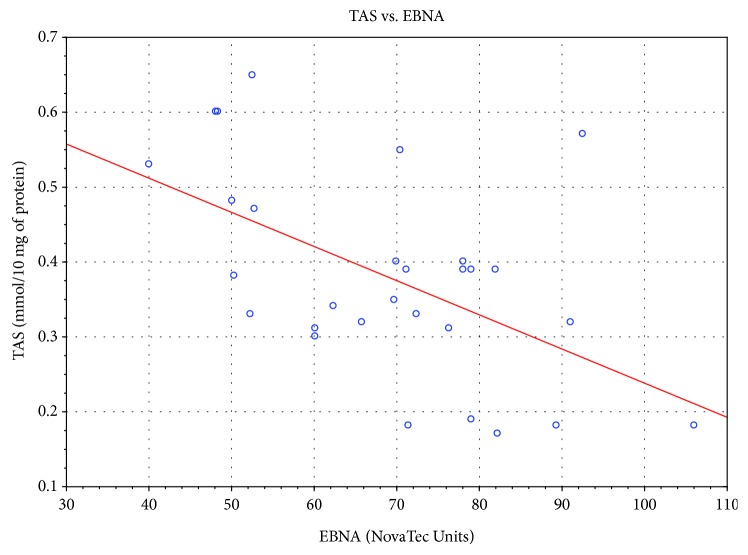
Correlation between tissue level of TAS and serum level of anti-EBNA. Spearman's rank correlation test; *r* = −0.469326; *p* = 0.0102.

**Figure 8:**
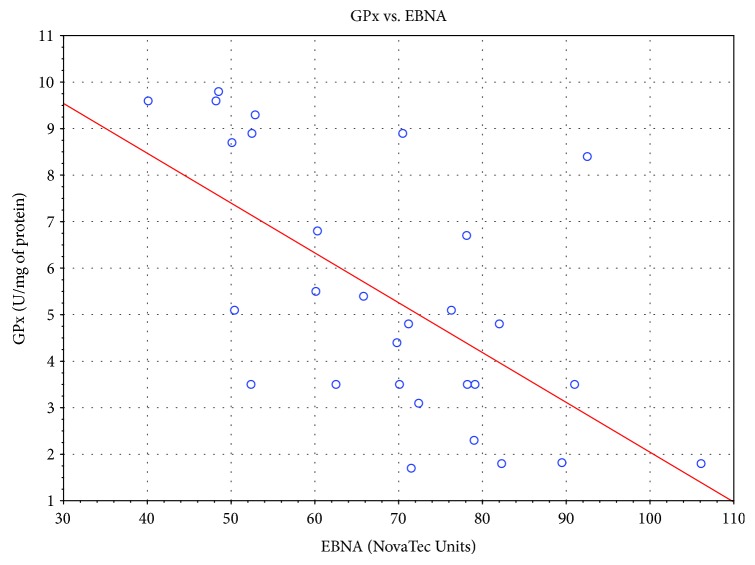
Correlation between tissue level of GPx and serum level of anti-EBNA. Spearman's rank correlation test; *r* = −0.651246; *p* = 0.0001.

**Figure 9:**
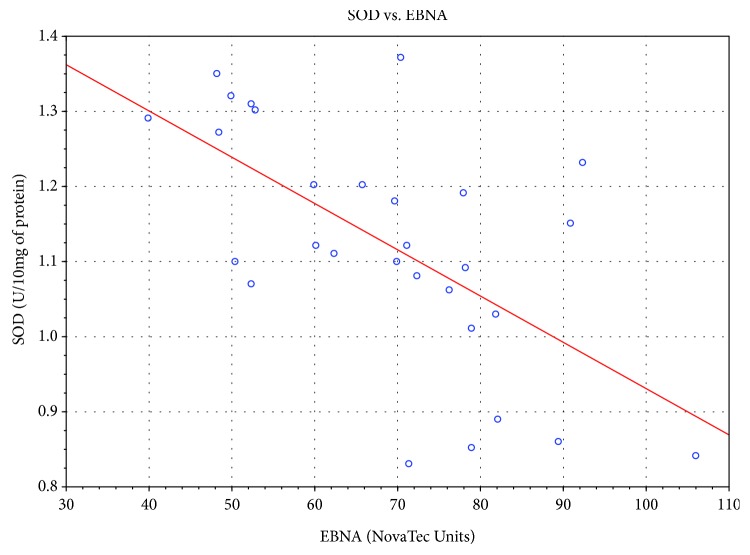
Correlation between tissue level of SOD and serum level of anti-EBNA. Spearman's rank correlation test; *r* = −0.6103034; *p* = 0.0004.

**Figure 10:**
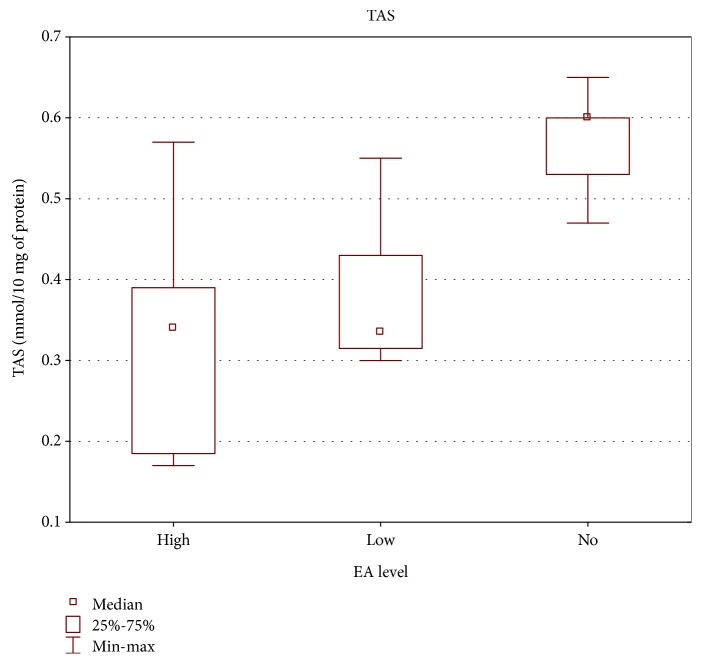
Correlation between tissue level of TAS and serum level of anti-EA. ANOVA rank Kruskal-Wallis test; *p* = 0.0057.

**Figure 11:**
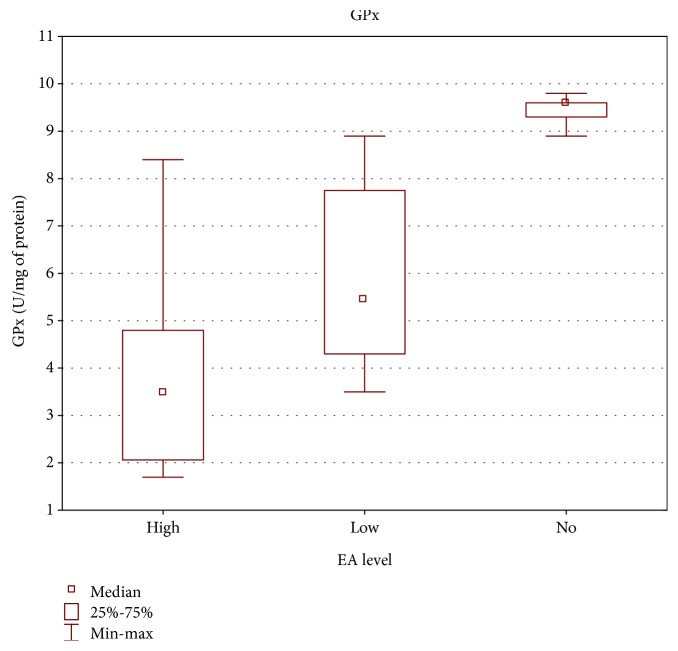
Correlation between tissue level of GPx and serum level of anti-EA. ANOVA rank Kruskal-Wallis test; *p* = 0.0003.

**Figure 12:**
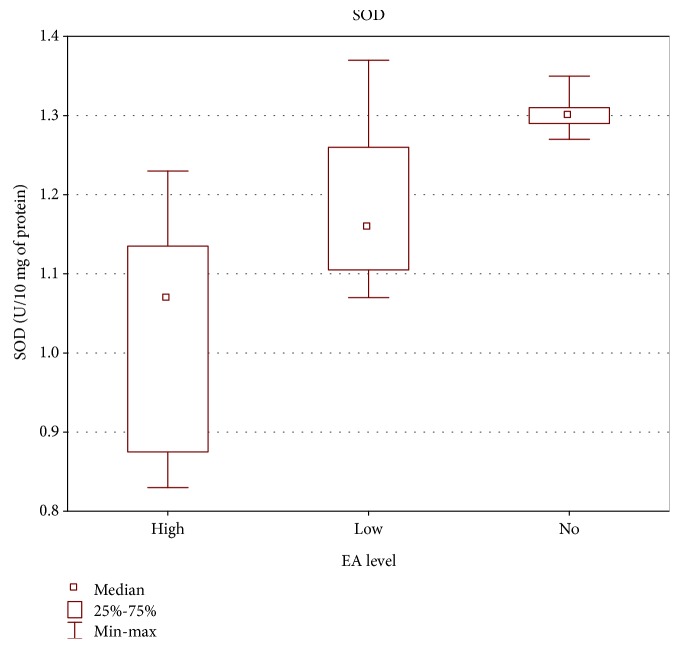
Correlation between tissue level of SOD and serum level of anti-EA. ANOVA rank Kruskal-Wallis test; *p* = 0.0010.

Significant correlation was also found between the level of specific antibodies against EBV and both TAS level and GPx and SOD activities. As the level of EBVCA and EBNA increased, the values of the tested parameters of oxidative stress decreased (*p* < 0.05 in all cases) (Figures 4–9). Figures 10–12 show a correlation between the tissue level of TAS, GPx, and SOD and the serum level of anti-EA. The analysis revealed the lowest values of oxidative stress parameters in high level of anti-EA (*p* < 0.05).

Values of oxidative stress parameters depended on histological grading: in poorly differentiated tumors (G3), the level of TAS and activities of GPx and SOD were significantly lower in EBV-positive patients than in EBV-negative (Table 5).

Differences were stated also in the values of oxidative stress parameters in different tumor dimensions (T), lymph node involvement (N) (Table 5). The activities of both GPx and SOD were significantly lower in T3-T4 than in T1-T2 among EBV-positive patients compared with EBV-negative. Similar difference was observed in the activities of GPx and SOD in different lymph node involvement (N).

## 4. Discussion

It is estimated that infection and chronic inflammation may contribute to about 25% of human cancers worldwide [27]. In such environment, inflammatory and epithelial cells generate ROS and reactive nitric species (RNS) and release cytokines, growth factors, which can cause DNA damage and alterations in critical pathways leading to cancer development or progression [28]. The extent of oxidative damage caused by ROS depends directly on antioxidant defense mechanism [29].

There are studies demonstrating increased oxidative stress and compromised antioxidant defenses in patients with oral cavity cancer [30]. Total antioxidant status (TAS) expresses the capacity for scavenging free radicals and reflects the residual antioxidant capacity after ROS neutralization [31]. The low activity of antioxidant enzymes plays an important role in the progression of lesion and leads to the development of oxidative stress [31, 32].

Our study revealed lower TAS level as well as decreased activities of SOD and GPx in cancer patients compared with the control group, similar to other research results [29, 31–36]. According to some authors, lower antioxidant enzyme activity might be caused by the depletion of antioxidant defense system occurring as the consequence of overproduction of free radicals [31, 32].

Opposite results were obtained by Bagul et al. [37]. They demonstrated statistically significant increase in GPx and SOD activities in OSCC patients compared with controls. Such outcomes may be explained by the fact that the patients in the initial stages of OSCC have high oxidative stress and lipid peroxidation. The level of free radicals may be higher, and the body tries to compensate it by increasing the level of antioxidants. Therefore, the increased serum activity of antioxidants might be a result of a natural defense mechanism to fight with carcinogenesis.

Moreover, SOD activity may be related with histological differentiation of tissues in various disorders. Rai et al. [29] analyzed the activity of this enzyme in patients with benign and malignant pathologies in the oral cavity. The mean SOD value was the highest in patients with oral leukoplakia and gradually decreased in oral submucous fibrosis, then in well-differentiated OSCC, with the lowest activity demonstrated in moderately differentiated OSCC. All differences were statistically significant. Results obtained by Gurudath et al. [19] and Singh et al. [31] are in concordance with the above outcomes as well as with our findings, which revealed similar tendency—both GPx and SOD values decreased with the lowering level of histological differentiation of tumor tissues. Singh et al. [31] in the study carried out in a group of patients with head and neck cancer demonstrated that poorly and moderately differentiated tumors were identified more frequently with lower TAS. They also found increased DNA damage in cancer patients and suppose that DNA damage may be related to insufficient antioxidant capacity and excessive ROS generation which contribute to the pathogenesis of cancer in HNC patients.

Decrease in antioxidant enzyme activity may be also related with advanced stage of tumor development. In the research performed by Srivastava et al. [33], lower values of all antioxidant enzymes (SOD, GPx, GSH, and CAT) were noted from stage II to stage IV (according to TNM) in oral cancer patients. Our study revealed similar correlation in the case of tumor dimensions and TAS and GPx values in EBV-negative patients, as well as in the case of lymph node involvement and TAS and antioxidant enzyme values both in EBV-positive and EBV-negative patients.

EBV is an important risk factor for human neoplasia associated with lymphoid and epithelial malignancies. Few studies, however, evaluated the correlation between viral infection, oxidative stress, and head and neck cancer.

According to Tsao et al. [38], EBV infection *per se* is not sufficient for tumorigenic transformation of epithelial cells. Some reports showed association between EBV infection and oxidative stress. Lassoued et al. [39] demonstrated that EBV infection of B cells and epithelial cells leads to oxidative stress which can play a crucial role during viral transformation. Bonner and Arbiser [40] proposed that similarly to Burkitt's lymphoma associated with EBV infection, other EBV-positive tumors may be also reactive oxygen tumors. Several EBV-encoded products such as LMP1, LMP2, and EBNA1 are associated with oxidative stress. Apart from initiating oncogenesis, they display also mechanisms of immune escape by interacting with and by modulating some immune-checkpoint inhibitors [41].

In several studies, it was demonstrated that EBV infection induces reactive oxygen, which may be sustained by the viral oncogene LMP1 and EBER and autocrine IL-10 production. Reactive oxygen signalling can be a characteristic of EBV-positive BL as increased levels of ROS are found in EBV-positive tumors, but not in EBV-negative tumors [40, 42].

Cerimele et al. [42] in a research carried out on different cell lines demonstrated that ROS was associated with EBV positivity. They not only found increased ROS in type I and type III latency but also noticed two mechanisms of EBV infection: in type 1 latency associated with absence of LMP1, EBER induced IL-10, which in turn induced ROS, while in type III latency, oncoprotein LMP1 induced ROS as a potential mechanism of cancer development. Antioxidant drug with superoxide dismutase and glutathione peroxidase-like activity completely inhibited ROS production. Moreover, they demonstrated that EBV-positive Burkitt's lymphoma cells used ROS as one of the major signalling pathways and these pathways were not activated in EBV-negative patients. Another important finding was that in type III latency EBNA2 target genes may stimulate ROS and that LMP1 was a major inducer of ROS in type III latency.

There are also studies indicating LMP1 as a possible ROS-inducing factor, but it was also demonstrated that the oxidative stress environment may affect the expression of LMP1 [16]. Moreover, LMP1 may be used in opposite ways by EBV in its life cycle. Expression of LMP1 may contribute to EBV lytic reactivation, but on the other hand, LMP1 may inhibit lytic cycle progression, inhibit EBV lytic reactivation, and may assist in establishing viral latency in B cells. Thus, EBV may use LMP1 for dual purposes in its life cycle [16].

Molecular studies demonstrated that a higher frequency of nasopharyngeal cancer detected in Asian population contains a variant of EBV *LMP1* gene with a 30 bp deletion (*del-LMP1*) [9, 43]. In our current research, in the majority of cases (82.3%), wild-type LMP1 was detected. Moreover, in patients infected with wt-LMP1, lower level of TAS and decreased activities of GPx and SOD were stated.

Other reports investigating EBV infection in human epithelial cells revealed that expression of LMP1 and LMP2 may induce stem cell properties in immortalized nasopharyngeal epithelial cells supporting a tumorigenic role of EBV infection. However, LMP1 is not detected in all NPC tumors and is not expressed at all in EBV-positive gastric cancer, which suggest that EBNA1 may play a more important role in the development of epithelial tumors [38, 44]. EBNA1 expressed in all types of EBV latency is the only EBV protein necessary for the persistence of EBV genome in latency and the only protein expressed in all EBV-positive tumors and sometimes the only protein expressed. It was found to change the cellular environment in various ways and as a result may contribute to cell immortalization and malignant transformation via interferences with tumor suppressors, induction of DNA damage, and altering of signalling pathways. It was documented that EBNA1 is expressed in all NPC tumors [38, 44].

Cao et al. [45] observed increased ROS levels in nasopharyngeal cancer cells expressing EBNA1 due to possible EBNA1-mediated transcriptional activation of NADPH oxidases. According to Kgatle et al. [46], cells infected with EBV induce DNA damage through the production of ROS caused by the activation of NOX and NADPH oxidase. It may lead to chronic infection and inflammation due to the activation of inflammasome triggering modifications of both viral and host genes crucial in the promotion of malignant transformation connected with EBV.

Kitagawa et al. [47] demonstrated the role of EBER in IL-10 induction. They found that EBV-positive cell clones expressed higher levels of IL-10 than EBV-negative subclones. Cerimele et al. [42], in turn, revealed that the inactivation of IL-10 led to the inhibition of ROS in type I latency BL. Our previous study is in agreement with these findings as increased level of different cytokines, including IL-10, was stated in patients with oropharyngeal cancer infected with EBV, indicating IL-10 involvement in the process of cancer development [48].

Our current research revealed higher level of antibodies against EBV including EA, EBVCA, and EBNA in patients with head and neck cancer when compared with controls, which may point to virus reactivation of latent EBV infection [13]. Moreover, the level of above antibodies was significantly higher in EBV-positive patients in comparison with EBV-negative ones and more than 50% of EBV-positive patients had high level of EA IgG. In our earlier research, all these antibodies were found in more than 90% of patients with oropharyngeal and laryngeal cancer and the levels of all these antibodies were higher in patients than in the control group and high levels were stated in about 80% of the cancer cases [22].

There are reports demonstrating that IgA antibodies are more useful and effective both in NPC screening tests and clinical diagnosis of this malignancy [8, 49, 50]. The current study analyzed only IgG antibodies. Immunoglobulins against EBV proteins such as EA-IgG, VCA-IgA, and Rta-IgG may be used as prognostic biomarkers in NPC. Tay et al. [12] state that EBV DNA load correlated with EA IgA serology titers may be useful in detection of early stages of NPC in screening tests.

Oxidative stress and agents causing damage to DNA have been demonstrated to induce the expression of EBV lytic genes. Reactivation of EBV can also be triggered by some chemical carcinogens implicated as risk factors of cancers associated with EBV, e.g., NPC. Carcinogens and EBV lytic infection synergistically increase oxidative stress, which is an integral link between environmental factors and EBV-associated cancers [51]. Arvey et al. [5] suggested that lytic reactivation of EBV takes place in oncogenesis of EBV-positive tumors and is a major risk factor for the development of disease related to EBV. In the process of EBV lytic reactivation, the virus encodes several antiapoptotic proteins, which usually play important roles in gaining resistance to apoptosis. Huang et al. [52] demonstrated in their study that mutagenic factor—N-methyl-N′-nitro-N-nitrosoguanidine (MNNG)—enhances the genomic instability and tumorigenicity of NPC cells through the induction of EBV reactivation. The mechanisms triggering EBV reactivation from latency, however, remain unclear. EBV reactivation was induced in more than 70% of EBV-positive NA cells (cell line) [52].

Studies performed in NPC patients revealed that EBV-specific proteins such as LMP1, LMP2, and EBNA1 may serve as possible target for vaccine development and immunological modulation [53]. In particular, EBNA1 and LMP2 have been demonstrated as attractive candidate vaccine targets due to their immunological competences and ability to cause latent infection [54].

Many recent studies have documented oxidative stress as a contributor to head and neck cancer. Our research is aimed at not only analyzing relationships between oxidative stress and oropharyngeal cancer but also investigating correlation between these two factors and EBV infection. A limitation of our study is, however, the small size of the studied group, especially of the del-LMP1 group, which makes statistical data comparing relationship between EBV type on the basis of the sequence in LMP1 gene and histological grading or TN stage not sufficiently strong. Disorders in antioxidant enzyme status balance may be considered not only a contributor to cancer development but also a possible biomarker and therapeutic target in cancer treatment strategy. Future studies are needed to understand the role of EBV lytic reactivation induced by oxidative stress. These biomarkers might have an important role in personalized therapy of EBV-related cancers.

## 5. Conclusions

The present study revealed lower level of TAS as well as decreased activities of antioxidant enzymes (GPx and SOD) in the tissue of the oropharyngeal cancer, particularly in EBV-positive patients. In majority (82.3%) of cases, wild-type LMP1 was detected. Significantly lower TAS, GPx, and SOD values were stated in patients infected with wild-type LMP1. The presence of anti-EA was detected in over 80% of patients, which suggests reactivation of EBV infection. The levels of examined parameters were the lowest during reactivation of EBV infection. The correlation between the degree of tumor differentiation and TN classification, especially in EBV-positive patients, was also stated. A weak antioxidant defense system may make the mucosal cells more vulnerable to the genotoxic effect of ROS. This creates an intracellular environment more prone to DNA damage and disease progression. Determination of these parameters may be useful in evaluating tumor burden in patients with various stages of oropharyngeal cancer and could be an important prognostic factor.

## Figures and Tables

**Table 1:** Clinical and epidemiological characteristics of patients.

		EBV				*p*	Total patients		Control group		*p*
		Positive		Negative							
		*n*	%	*n*	%		*n*	%	*n*	%	
Sex	Female	3	10.3	4	10.8	0.9513	7	10.6	3	10.0	>0.05
	Male	26	89.7	33	89.2		59	89.4	27	90.0	

Age	<50	2	6.9	7	18.9	0.1288	9	13.6	4	13.3	>0.05
	50-69	21	72.4	18	48.7		39	59.1	18	60.0	
	70+	6	20.7	12	32.4		18	27.3	8	26.7	

Place of residence	Urban	25	86.2	26	70.3	0.1251	51	77.3	23	76.7	>0.05
	Rural	4	13.8	11	29.7		15	22.7	7	23.3	

Smoking	Yes	18	62.1	23	62.2	0.6641	41	62.1	19	63.3	>0.05
	No	11	37.9	14	37.8		25	37.9	11	36.7	

Alcohol abuse	Yes	13	44.8	16	43.2	0.8976	29	43.9	13	43.3	>0.05
	No	16	55.2	21	56.8		37	56.1	17	56.7	

G	G1	8	27.6	15	40.5	0.4941	—		—		—
	G2	16	55.2	18	48.7						
	G3	5	17.2	4	10.8						

T	T1-T2	18	62.1	24	64.9	0.8147	—		—		—
	T3-T4	11	37.9	13	35.1						

N	N1-N2	20	68.9	25	67.6	0.0942	—		—		—
	N3-N4	9	31.1	12	32.4						

M	M0	29	100.0	37	100.0	—	—		—		—

LMP-1	wt-LMP-1	24	82.3	—	—	—	—		—		—
	del-LMP-1	5	17.7	—	—						

Pearson's chi-square test.

**Table 2:** TAS level (mmol/10 mg of protein) and GPx (U/mg of protein) and SOD (U/10 mg of protein) activities in the tissue of EBV-positive and EBV-negative patients in comparison with the control group.

	Patients ($\bar{x}\pm \text{SD}$)		Controls ($\bar{x}\pm \text{SD}$)	*p* value^2^
	EBV+	EBV-		
TAS	0.38 ± 0.13	0.66 ± 0.47	0.81 ± 0.19	10^−5^^∗^
*p* value^1^	0.0173^∗^			
GPx	5.36 ± 2.7	10.85 ± 2.8	17.36 ± 1.7	10^−6^^∗^
*p* value^1^	10^−6^^∗^			
SOD	1.12 ± 0.16	1.81 ± 0.14	2.63 ± 0.22	10^−6^^∗^
*p* value^1^	10^−6^^∗^			

^∗^Statistically significant; 1: Mann-Whitney *U* test; 2: ANOVA Kruskal-Wallis test.

**Table 3:** TAS level (mmol/10 mg of protein) and GPx (U/mg of protein) and SOD (U/10 mg of protein) activities in cancer tissue of patients infected with wild-type EBV (wt-LMP-1) in comparison with del-LMP-1.

	TAS ($\bar{x}\pm \text{SD}$)	GPx ($\bar{x}\pm \text{SD}$)	SOD ($\bar{x}\pm \text{SD}$)
wt-LMP-1	0.33 ± 0.1	4.3 ± 1.9	1.07 ± 0.13
del-LMP-1	0.57 ± 0.06	9.4 ± 0.4	1.31 ± 0.03
*p* value	0.0005^∗^	4 × 10^−6∗^	0.0005^∗^

^∗^Statistically significant; Mann-Whitney *U* test.

**Table 4:** Serum antibody level in EBV-positive and EBV-negative patients in comparison with the control group (NTU: NovaTec-Units).

	Patients ($\bar{x}\pm \text{SD}$)				Controls ($\bar{x}\pm \text{SD}$)		*p* value^2^
	EBV+		EBV-				
EBVCA	72.9 ± 17.7		63.4 ± 9.6		66.8 ± 12.7		0.0198^∗^
*p* value^1^	0.0107^∗^						
EBNA	69.1 ± 15.8		60.8 ± 9.4		56.3 ± 10.2		0.0031^∗^
*p* value^1^	0.0285^∗^						

EA	*N*	%	*N*	%	*N*	%	10^−6^^∗^
High level	16	55.1	0	0	0	0	
Low level	8	26.7	10	27.03	12	40.0	
No EA	5	17.2	27	72.97	18	60.0	

^∗^Statistically significant; EBVCA, EBNA: 1: Mann-Whitney *U* test; 2: ANOVA Kruskal-Wallis test; EA: Pearson's chi-square test.

**Table 5:** Comparison between tissue levels of TAS, activities of GPx and SOD, and G, T, N in EBV-positive and EBV-negative patients with oropharyngeal cancer.

	TAS		*p* value	GPx		*p* value	SOD		*p* value
	EBV+	EBV-		EBV+	EBV-		EBV+	EBV-	
G1	0.56 ± 0.06	1.04 ± 0.53	0.0707	9.15 ± 0.50	13.93 ± 0.65	0.0001^∗^	1.31 ± 0.04	1.91 ± 0.12	0.0001^∗^
G2	0.35 ± 0.04	0.39 ± 0.15	0.8495	4.54 ± 1.18	9.32 ± 0.74	10^−6^^∗^	1.11 ± 0.06	1.80 ± 0.03	10^−6^^∗^
G3	0.18 ± 0.007	0.43 ± 0.06	0.0199^∗^	1.88 ± 0.24	6.23 ± 0.63	0.0199^∗^	0.85 ± 0.02	1.52 ± 0.05	0.0199^∗^
T1-T2	0.33 ± 0.13	0.80 ± 0.52	0.0004^∗^	4.68 ± 2.53	11.29 ± 3.19	10^−6^^∗^	1.07 ± 0.16	1.82 ± 0.17	10^−6^^∗^
T3-T4	0.45 ± 0.11	0.40 ± 0.17	0.2129	6.46 ± 2.80	10.05 ± 1.83	0.0077^∗^	1.20 ± 0.11	1.82 ± 0.06	3 × 10^−6∗^
N1-N2	0.40 ± 0.14	0.92 ± 0.61	0.0527	5.60 ± 2.75	11.14 ± 2.52	6 × 10^−5∗^	1.14 ± 0 .15	1.84 ± 0.12	2 × 10^−6∗^
N3-N4	0.34 ± 0.13	0.50 ± 0.27	0.0742	4.96 ± 2.79	10.88 ± 3.05	0.0001^∗^	1.09 ± 0.17	1.81 ± 0.15	5 × 10^−6∗^

^∗^Statistically significant; Mann-Whitney *U* test.

## Data Availability

The data used to support the findings of this study are included within the article.
